# The potential economic benefits of controlling trypanosomiasis using waterbuck repellent blend in sub-Saharan Africa

**DOI:** 10.1371/journal.pone.0254558

**Published:** 2021-07-20

**Authors:** Zewdu Abro, Menale Kassie, Beatrice Muriithi, Michael Okal, Daniel Masiga, Gift Wanda, Ouedraogo Gisèle, Abah Samuel, Etienne Nguertoum, Rock Aimé Nina, Philémon Mansinsa, Yahaya Adam, Mamadou Camara, Pamela Olet, Diarra Boucader, Susana Jamal, Abdoul Razak Issa Garba, Joseph Joachim Ajakaiye, Jean Felix Kinani, Mohamed Adam Hassan, Hezron Nonga, Joyce Daffa, Ambrose Gidudu, Kalinga Chilongo

**Affiliations:** 1 International Centre of Insect Physiology and Ecology (*icipe*), Addis Ababa, Ethiopia; 2 International Centre of Insect Physiology and Ecology (*icipe*), Nairobi, Kenya; 3 African Union, Coordinator of the Pan *African* Tsetse and Trypanosomiasis (T&T) Eradication Campaign (PATTEC), Addis Ababa, Ethiopia; 4 Country Coordinators of the Pan *African* Tsetse and Trypanosomiasis (T&T) Eradication Campaign (PATTEC) for Burkina Faso, Cameroon, Central Africa Republic, Congo, Democratic Republic of the Congo, Ghana, Guinea, Kenya, Mali, Mozambique, Niger, Nigeria, Rwanda, Sudan, Tanzania, Tanzania, Uganda, and Zambia, respectively; University of Zambia, ZAMBIA

## Abstract

Trypanosomiasis is a significant productivity-limiting livestock disease in sub-Saharan Africa, contributing to poverty and food insecurity. In this paper, we estimate the potential economic gains from adopting Waterbuck Repellent Blend (WRB). The WRB is a new technology that pushes trypanosomiasis-transmitting tsetse fly away from animals, improving animals’ health and increasing meat and milk productivity. We estimate the benefits of WRB on the production of meat and milk using the economic surplus approach. We obtained data from an expert elicitation survey, secondary and experimental sources. Our findings show that the adoption of WRB in 5 to 50% of the animal population would generate an economic surplus of US$ 78–869 million per annum for African 18 countries. The estimated benefit-cost ratio (9:1) further justifies an investment in WRB. The technology’s potential benefits are likely to be underestimated since our estimates did not include the indirect benefits of the technology adoption, such as the increase in the quantity and quality of animals’ draught power services and human and environmental health effects. These benefits suggest that investing in WRB can contribute to nutrition security and sustainable development goals.

## Introduction

Trypanosomiasis is a significant challenge for livestock health and economic performance in sub-Sahara Africa (SSA) [1]. It is caused by the trypanosome parasite that causes nagana in domesticated animals and sleeping sickness in humans. Livestock production plays an important economic and socio-cultural role in the livelihoods of rural households, such as food and nutrition supply, and source of draught power, employment income, soil fertility, and capital accumulation. Livestock production accounts for 40% of total household income across all livestock production systems in SSA [2]. The region also houses nearly 309 million livestock keepers who live below US$ 2 per day [2]. The dependence of farmers on livestock for livelihood makes them vulnerable to various diseases, including trypanosomiasis. Estimates show that 32% of the SSA’s livestock population are found in tsetse-fly-infested areas where the threat varies by region [3]. Of the total cattle population, 48%, 76%, 28%, and 8% of the animals are at risk of trypanosomiasis in Western Africa, Central Africa, Eastern Africa, and Southern Africa, respectively [3].

Trypanosomiasis reduces milk and meat production and income [4]. It indirectly affects land use by reducing the draught power productivity of oxen. Because of the risk of trypanosomiasis, farmers avoid productive tsetse-fly-infested areas, which might need to be used in the face of high population growth [5–10]. It is estimated to cause 3 million deaths of cattle with an annual direct economic loss of US$ 1–1.2 billion in cattle production. Accounting for indirect economic losses, SSA may lose up to 4.75 billion of GDP per year [9–13].

Achieving the Sustainable Development Goals (SDGs) needs to address the livestock sector’s constraints, one of the key sources of livelihood for poor people [14]. Given that SSA is one of the world’s regions where food insecurity and malnutrition are widespread, controlling trypanosomiasis is one potential avenue to improve livelihood [15]. To avoid economic losses from trypanosomiasis and further increase the livestock sector’s productivity, the African Union (AU) established the Pan African Tsetse and Trypanosomiasis Eradication Council (PATTEC) in 2000. Through the PATTEC, the AU envisions a continent with no trypanosomiasis. It assists countries by providing financial, policy, and institutional support to reduce the impact of trypanosomiasis [16]. Multilateral institutions, such as the Food and Agriculture Organization (FAO), collaborate with the AU by providing technical and financial support to eradicate the disease [5, 17–20]. Even though these institutions spent a significant amount of financial and non-financial resources, trypanosomiasis remains a threat to SSA. The threat of trypanosomiasis may be further worsened by climate change as it may increase the incidence of tsetse fly in traditionally non-tsetse fly areas [21–23]. These existing challenges call for innovations that address the needs of the poor livestock farmers and global environmental sustainability.

Various tsetse fly and trypanosomiasis controlling techniques have been used in the past [1, 6, 10, 24]. Treating sick animals using trypanocides is one of the most commonly used approaches [8, 25–28]. Preventive measures that target the tsetse fly vector itself are widely used in various communities. These measures include clearing the habitat of the tsetse fly, using baited targets, sequential aerosol technique (SAT), ground spraying, insecticide-treated cattle (ITC), and sterile insect technique (SIT) [6–8, 29]. Despite the initial successes of using these approaches, with few exceptions, SSA has not still been able to control trypanosomiasis [30].

Implementers of the control techniques on the ground and African livestock policymakers face several challenges [31]. Drug resistance is one of the challenges facing livestock producers [32]. Low quality and counterfeit drugs further worsen the problem of resistance to trypanocides [6, 25, 33]. Even though rearing trypanotolerant breed of animals seems promising, the proportion of animals with the required level of resistance is small, and importing these breeds to other production contexts proved difficult [9, 29]. The financial sustainability of some of the techniques (e.g., aerial or ground spraying, SAT, and SIT) has been questioned [6, 9]. Clearing forests and vegetation, killing game animals, and pesticide spraying are not environmentally friendly [8, 29]. They are not compatible with the SDGs, emphasizing balancing development outcomes and environmental sustainability [14]. Furthermore, climate change consequences are already enormous, and biodiversity loss is a pressing problem worldwide [34]. These emerging concerns are the fundamental driving forces for developing the waterbuck repellent blend (WRB) [5, 35, 36].

**Fig 1:**
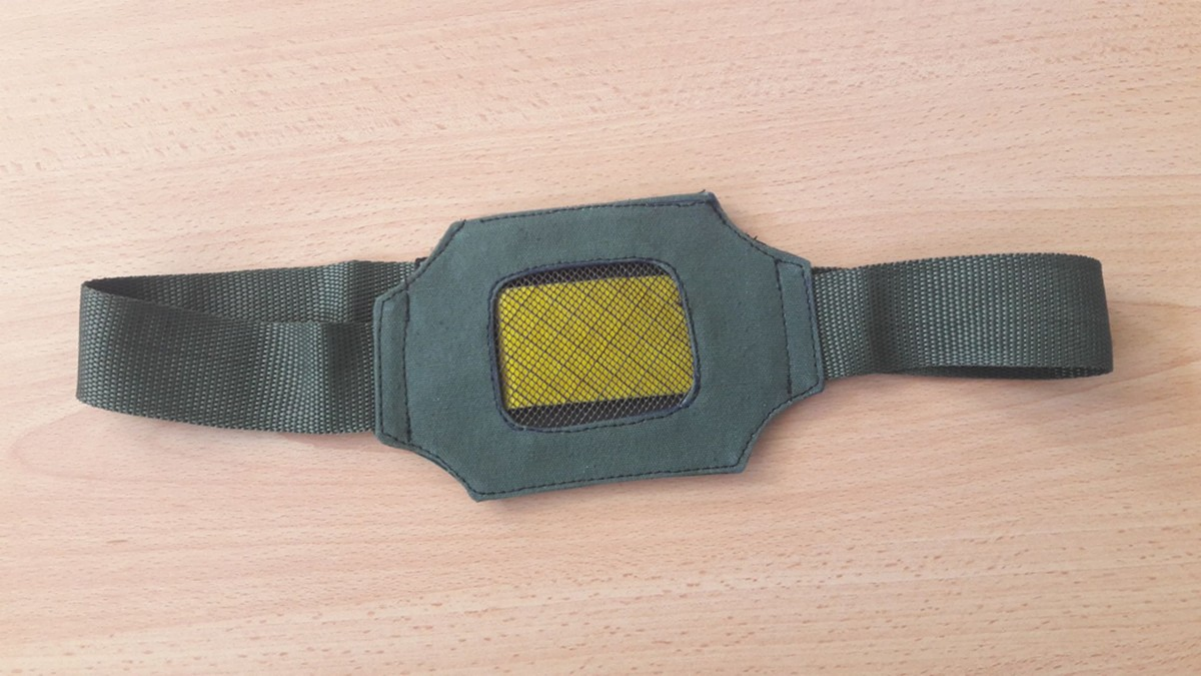
Tsetse fly WRB collar.

The technology incorporates a collar dispenser and the distinct chemical odors that push tsetse flies away from animals (WRB), reducing exposure to infection significantly [5]. The WRB is a chemical blend initially identified from waterbucks that repel tsetse fly [5]. The technology, developed by the International Centre of Insect Physiology and Ecology (*icipe*), tested its effectiveness, and modified it several times. Fig 1 shows the tsetse fly WRB collar. Farmers must change the collar every six months and WRB every six weeks. Farmers incur all the investment costs in less than a year, and they harvest benefits within one year. The WRB control tsetse fly and trypanosomiasis effectively and sustainably [5]. The WRB is novel in the sense that it is eco-friendly because it does not affect beneficial insects. The increased draught power capacity has been shown to lead to an increase in land cultivated and earnings from oxen rental in tsetse-infested areas [5, 37]. Reduced animal mortality rates and increased milk production, improving food and nutrition security. The WRB is a ready-made technology that can easily be tied to the neck of the animals. Unlike most of the available tsetse fly control techniques, farmers can use the technology themselves with no expert knowledge. Another attractive feature of the technology is that it is scalable because it is cheap and easily movable. Given these attractive features of the technology, it is essential to estimate its potential benefits to foster its upscaling in SSA.

This paper demonstrates the potential economic benefits of WRB using data from expert opinions from 18 countries in Central, Eastern, Northern, and Western Africa and experimental and secondary sources. We contribute to the literature in the following ways. First, to our knowledge, we are the first to quantify the *ex-ante* potential economic benefits of adopting WRB in SSA, important for informed livestock health investment and promoting the technology. Second, the study adds evidence on the limited studies on existing trypanosomiasis control efforts and their welfare implications [6, 8, 10]. Third, the study contributes to the various initiatives, including but not limited to the PATTEC and FAO’s Programme Against African Trypanosomiasis (PAAT). Fourth, the research can inform land-use planners to decide on using land abandoned due to trypanosomiasis risk.

## Materials and methods

### Data sources and data collection methods

We rely on three data sources. First, we use data collected through an expert elicitation survey facilitated by the African Union–Pan African Tsetse and Trypanosomiasis Eradication Campaign (AU-PATTEC) and the International Centre of Insect Physiology and Ecology (*icipe*). The survey had an introduction page that explains the survey objective to PATTEC focal persons (experts). A wide range of data collected using the expert elicitation method. These include (1) the prevalence and percentage of animals at risk of trypanosomiasis, (2) mortality rate, (3) trypanosomiasis impacts on meat and milk production, (4) annual expenditure to control the disease, (5) total area infested, and cultivated land abandoned due to the disease; (6) experts’ current position and expertise, (7) the number of years of experience working with the livestock sector, and (8) the number of years working in control of tsetse and trypanosomiasis. We also collected data on current trypanosomiasis control measures in each country. The questionnaire was dispatched to 39 SSA countries where tsetse fly and trypanosomiasis is a problem. The countries were Angola, Benin, Botswana, Burkina Faso, Burundi, Cameroon, Central Africa Republic (CAR), Chad, Republic of the Congo, Democratic Republic of the Congo (DRC), Cote d′ivoire, Equatorial Guinea, Ethiopia, Gabon, Gambia, Ghana, Guinea, Guinea Bissau, Kenya, Liberia, Malawi, Mali, Mozambique, Namibia, Niger, Nigeria, Rwanda, Senegal, Sierra Leone, Somalia, South Africa, South Sudan, Sudan, Swaziland, Tanzania, Togo, Uganda, Zambia, and Zimbabwe. But we obtained responses from 18 countries only: Burkina Faso, Cameroon, Central Africa Republic, Republic of the Congo, Democratic Republic of the Congo, Ethiopia, Ghana, Guinea, Kenya, Mali, Mozambique, Niger, Nigeria, Rwanda, Sudan, Tanzania, Uganda, and Zambia.

Second, we make use of the various datasets in FAOSTAT [38]. FAOSTAT is the critical data source on a country-level number of animals, meat and milk production, price of meat and milk products, and the livestock sector’s contribution to agricultural production. Third, we rely on the literature to fill the remaining data gaps. The elasticity of supply and demand for animal products and potential productivity gains and cost reductions associated with the WRB are gathered from the literature, which will be discussed below. We analyzed the data using Stata software. We provided the steps followed and the Stata codes used for data analysis in the (S1 File). We also provided the final datasets and the estimated benefits of the technology in the (S2 File).

### Estimation of economic surplus

This section describes the methods and steps to estimate the potential economic surplus of introducing WRB in SSA. This paper aims to assess the meat and milk economic surplus that could be obtained by adopting WRB. Other potential benefits of the technology are not quantified (including animal draught power productivity and human health effects) because of a lack of data.

We quantify the potential economic surplus of adopting WRB using the economic surplus model (ES) [39]. New livestock production technology, in our case, WRB, will shift the supply curve for livestock products. This will directly influence producers by changing production costs and productivity. It indirectly affects consumers due to changes in the prices of meat and milk. The benefit of the technology to producers and consumers may depend on the type of markets assumed. In the absence of external trade (a closed economy), the technology’s benefit is shared between producers and consumers. The assumption of a closed economy seems plausible in the context of SSA because the countries in this region have no or little international trade on meat (camel, cattle, goats, pigs, and sheep) and milk (cows and camel), perhaps with a small impact on domestic prices. Export of meat and milk is only 1% and 0.55% of the domestic production, respectively. The import of meat is limited to 7% of the domestic meat supply, while milk is limited to 1% [38].

Because the WRB is a new technology, we quantify its *ex-ante* impact on the consumer (ΔCS) and producer (ΔPS) surpluses changes in two steps. In the first step, we estimate K-shift parameters of meat and milk, which are the WRB -induced proportionate shift in the supply curves per unit of production cost reduction [39]. The K-shift parameters for meat and milk are defined in Eq (1).

$$K_{m}=\left(\frac{{ATT}_{ym}}{\epsilon_{m}}-\frac{{ATT}_{cm}}{1+{ATT}_{ym}}\right)\times r$$ (1)

where the index *m* stands for livestock products: meat and milk. ATT_ym_ and ATT_cm_ represent the proportionate change in the productivity and cost of production of product *m* due to the introduction of WRB. The parameters ATT_ym_ for meat and milk are respectively 50% and 51% [3, 4]. These figures imply that controlling trypanosomiasis could increase meat and milk productivity by 50 and 51%, respectively. In addition to the WRB’s productivity benefits, a field experiment in Kenya shows that WRB could reduce the cost of livestock production by 153% [5]. We take this as the estimated value of ATT_cm_. The price elasticity of supply (*ϵ*_*m*_) is 0.40, which is the average long-run aggregate supply elasticity of agriculture in SSA [40]. Despite that the supply elasticity is an aggregate value to agriculture, it seems reasonable to use it for livestock products, which definitely shows that the livestock sector is slow to respond to price because it takes several years to build herds of animals [41].

The K-shift parameters in Eq (1) are weighted by the potential adoption rate (*r*) of WRB by the livestock industry in each country. No actual data on the adoption rate exists because the WRB is new. Therefore, in our benefits estimation, we assume various adoption rates of WRB in the livestock sector: 5%, 15%, 25%, and 50%. We believe that WRB’s immediate sensitization could lead to adoption rates of 5% to 15% of the animals. Complete transition to using the technology may need not only convincing farmers but actors that commercialize the technology. The adoption rate of WRB of 5 to 15% of the animals may reflect the benefits that could potentially accrue in the short run. In the long-run, the supply and demand-side constraints may be relaxed through awareness creation, capacity building, policy dialogue, and learning by providing more evidence on the technology’s cost and benefits to farmers, livestock policymakers, and agro-dealers [42]. Adoption rates of 25 to 50% may indicate the WRB’s long-run benefits.

In the second step, we estimate the change in total economic benefits that accrue to consumers and producers of meat and milk because of WRB’s adoption. The changes in producer surplus (ΔPS) and consumer surplus (ΔCS) under a closed economy attributed to the adoption of WRB are as defined in Eqs (2) and (3) [39].

$${\Delta PS}_{m}=P_{m}Q_{m}(K_{m}-Z_{m})(1+0.5Z_{m}\eta_{m})$$ (2)

$${\Delta CS}_{m}=P_{m}Q_{m}Z_{m}(1+0.5Z_{m}\eta_{m})$$ (3)

where *P*_*m*_ and *Q*_*m*_ are the average producer price and quantity of product *m* before the introduction of the WRB; Z_m_ is the relative change in price *P*_*m*_ (*Z*_*m*_ = *K*_*m*_×*ϵ*_*m*_/(*ϵ*_*m*_+*η*_*m*_)) [39]; *η*_*m*_ is the absolute price elasticity of demand for product *m*, which varies by country, obtained from the United States Department of Agriculture Economic Research Service [43]. Adding Δ*PS*_*m*_ and Δ*CS*_*m*_ provides the total potential economic surplus (ES) that could be generated from adopting WRB.

It is worth noting that the baseline information on *P*_*m*_ and *Q*_*m*_ are crucial in the calculation of the economic surplus. The FAOSTAT database does not have disaggregated data on meat and milk production specific to tsetse-fly-infested areas. The baseline meat and milk production that might be affected by trypanosomiasis is given by Eq (4).

$$Q_{m}=(\pi \times \gamma \times N_{m})\times Y_{m}$$ (4)

where the multiplicative term *π*×*γ*×*N*_*m*_ provides the number of meat and milk animals potentially affected by trypanosomiasis in tsetse-fly-infested areas. *π* and *γ* are the percentages of animals at risk, and the prevalence rate of trypanosomiasis, respectively. The estimates of *π* and *γ* are obtained from the expert opinion survey discussed in the previous section. *N*_*m*_ stands for the number of animals that produce product *m*, while *Y*_*m*_ is the productivity of product *m* (tonnes/animal), which is 50% lower than the productivity of animals under normal circumstances [4]. Table 1 summarizes some of the parameters used to estimate Eqs (2–4).

**Table 1:** Parameters of the economic surplus model.

Parameters	Mean	Standard deviations	minimum	maximum	Total
Number of meat animals affected by trypanosomiasis	5,528,327	6,774,660	119,921	25,874,000	99,509,886
Number of milk animals affected by trypanosomiasis	8,116,138	12,964,231	2,860	50,038,380	146,090,482
Prevalence rate of trypanosomiasis (%)	14	11	1	47	NA
% of animals at risk of trypanosomiasis	33	12	4	49	NA
Meat productivity (tonnes/head)	0.11	0.10	0.04	0.44	NA
Milk productivity (tonnes/head)	0.41	0.45	0.05	1.39	NA
Meat production (tonnes)	7,306	6,404	51	19,600	131,514
Milk production (tonnes)	23,254	36,402	44	133,485	418,576
Elasticity of demand for meat	-0.59	0.01	-0.62	-0.56	NA
Elasticity of demand for milk	-0.61	0.01	-0.64	-0.58	NA
Elasticity of supply for meat	0.40	NA	0.40	0.40	NA
Elasticity of supply for milk	0.40	NA	0.40	0.40	NA
Price of meat (US$/tonnes)	3,235	1,638	1,387	5,304	NA
Price of milk (US$/tonnes)	681	159	344	944	NA

Note: (1) The prices and quantity parameters except the elasticity are five years averages (2013–2017) of each country; We have used the five-year average because it helps to smooth out shock-induced fluctuations in meat and milk production and associated prices;(2) The mean data for the number of meat and milk animals affected, and meat and milk production are displayed to summarize the countries average ownership, but in the analysis, we have used the actual numbers in each country; (3) NA refers not applicable.

### Estimation method for benefit-cost ratio

It might be important to understand whether an investment in promoting WRB is worthwhile. This could be done by comparing the economic surplus discussed in the previous section with the technology costs. Given that the technology is new, finding cost data is difficult. However, in Kenya, the WRB could cost farmers 30 US$/cattle head/year [44]. However, this unit cost of WRB may not apply to other countries because of the difference in transaction costs. To account for transaction costs, we uniformly impose a 5% increase in the cost of adoption. Eq (5) below calculates a benefit-cost ratio (*BCR*), which shows the additional gains of a one-dollar investment on WRB.

$$BCR=\frac{ES}{N\times CRCT}$$ (5)

where ES = Δ*PS*_*m*_+Δ*CS*_*m*_, *N* is the number of meat and milk animals at risk, and *CRCT* is the unit cost of adopting WRB. A *BCR* greater than one justifies an investment in WRB.

## Results and discussion

This section presents findings from the experts’ opinions survey, the economic surplus model, and the benefit-cost ratio of adopting WRB.

### Results from the expert elicitation survey

Table 2 presents the responses of the key variables obtained from the experts’ survey. On average, 33% of the animals in these countries are at risk of trypanosomiasis (Table 2). The reported prevalence rate is 14%, much closer to the 15% prevalence rate of trypanosomiasis in 19 countries in SSA [31]. Furthermore, the potential impact of trypanosomiasis on productivity losses is high, reducing meat and milk productivity by 36% and 34%. Closing the trypanosomiasis-induced productivity losses would contribute to reducing malnutrition in these countries [45].

**Table 2:** Economic burden of Trypanosomiasis disease reported by experts.

	Indicators (%)		Productivity loss due to trypanosomiasis		Yearly expenditure (‘000 US$)
Country	Animals at risk	Prevalence rate	Meat	Milk	
Burkina Faso	35	7	30	40	NI
Cameroon	39	15	30	26	166
Central Africa Republic	36	47	75	60	8,997
Republic of the Congo	33	33	40	10	14,396
Democratic Republic of the Congo (DRC)	18	5	89	19	1
Ethiopia ^**¥**^	20	8	NI	NI	NI
Ghana	47	9	NI	NI	119
Guinea	41	9	5	10	127
Kenya	46	12	20	20	11,846
Mali	17	20	NI	NI	54
Mozambique	32	10	NI	NI	NI
Niger	4	1	1	1	9
Nigeria	30	9	41	62	889
Rwanda	37	6	1	1	NI
Sudan	49	9	25	75	4,890
Tanzania	35	10	90	86	66
Uganda	28	20	NI	NI	939
Zambia	46	15	22	33	1,195
**Average**	**33**	**14**	**36**	**34**	**43,693**

Source: Experts’ opinion survey;

^**¥**^ Unlike other countries where the data obtained from the experts’ opinions survey, Ethiopia’s data was obtained from Leta et al. 2016 [7]. NI refers to no information available.

Besides the productivity losses, the fiscal cost of trypanosomiasis is high. On average, the governments in the study countries spend US$ 44 million per annum to control trypanosomiasis (Table 2). If countries can use new technologies such as the WRB, they could use this expenditure for other development activities. The Republic of Congo, Kenya, Central Africa Republic, Uganda, and Nigeria invest 85% of all countries’ total spending on trypanosomiasis control. Furthermore, the data suggest that some countries may not have enough resources to control and eradicate trypanosomiasis, although the disease’s risk is significant. For instance, the reported risk of trypanosomiasis and its impact on productivity is high in Cameroon and the Democratic Republic of the Congo (DRC). Still, their expenditure is low relative to other countries. Some countries, for instance, The Republic of the Congo, reported relatively higher government expenditure for trypanosomiasis control and eradication compared to the gains from the WRB. However, trypanosomiasis prevalence remains huge, indicating higher expenditure does not necessarily translate into trypanosomiasis reduction [30].

### Results of the economic surplus model

**Fig 2:**
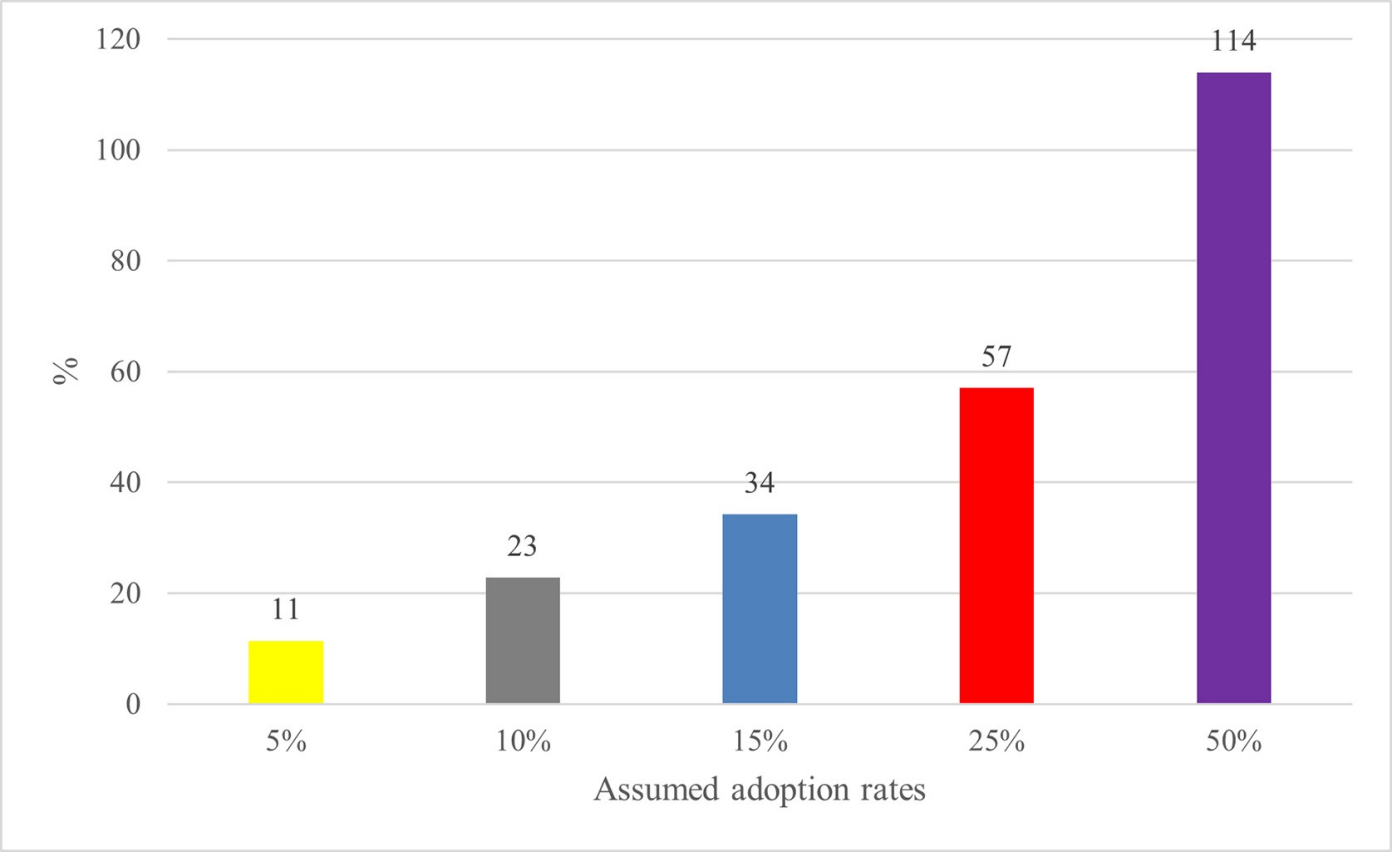
K-shift parameters (%).

We start presenting our results by showing the estimated K-shift parameter in Fig 2. In the short-run, a 5−15% adoption of WRB in the livestock sector could reduce the cost of meat and milk production by 11–34% regardless of the type of animal products. This could be an essential contribution to the countries’ economy where their meat and milk productivity is affected by trypanosomiasis, which will have aggregate significant economic benefits. The k-shift parameter substantially increases by 57% and 114% if the countries adopt WRB at the adoption rates of 25 and 50%.

In Table 3, we report the economic surplus that producers and consumers of animal products could potentially obtain if producers of meat and milk adopt WRB. In the short run, a 5 to 15% adoption of WRB would generate an economic surplus that reaches US$ 78 to 239 million per annum (Table 3). In the long-term, the total economic benefits could reach US$ 409 and 869 million per annum if the rate of adoption of WRB increases to 25 and 50%, respectively. The estimated economic surplus is far greater than the expenditure on trypanosomiasis control and eradication for most countries. Zambia, for instance, spends US$ 1.2 million (Table 2) for trypanosomiasis control and eradication per annum, which is nearly three-fourth of the estimated benefits of WRB at a 5% adoption rate. An exception to this is the Republic of the Congo that spends more money than the economic surplus. Table 3 further reveals that 68% of the economic surplus comes from gains in meat production. Producers of meat and milk take nearly 60% of the benefits, while 41% go to consumers.

**Table 3:** Estimated economic surplus from WRB adoption (millions of US$).

	Adoption rates						Producer surplus (%)
Country	5%	10%	15%	25%	50%	Meat Economic surplus (%)	
Ethiopia	3.53	7.15	10.87	18.59	39.59	39	61
Kenya	15.13	30.67	46.62	79.72	169.59	35	60
Mozambique	1.36	2.75	4.18	7.15	15.21	52	60
Rwanda	0.49	0.99	1.51	2.58	5.48	56	60
Uganda	6.27	12.70	19.30	33.02	70.25	47	60
Tanzania	4.29	8.69	13.21	22.59	48.08	44	60
Zambia	3.37	6.82	10.37	17.73	37.71	71	60
**Sub-total: Eastern Africa**	**34**	**70**	**106**	**181**	**386**		
Cameroon	1.86	3.76	5.72	9.78	20.80	57	59
CAR	3.06	6.20	9.42	16.11	34.27	76	60
Republic of the Congo	0.49	1.00	1.52	2.61	5.54	95	59
Democratic Republic of the Congo (DRC)	0.17	0.35	0.53	0.90	1.92	97	61
**Sub-total: Central Africa**	**5.58**	**11.31**	**17.19**	**29.40**	**62.53**		
**North Africa: Sudan**	**15.62**	**31.66**	**48.10**	**82.22**	**174.72**	**75**	**59**
Burkina Faso	1.76	3.57	5.43	9.29	19.76	81	60
Ghana	2.51	5.09	7.74	13.23	28.13	98	59
Guinea	1.42	2.89	4.39	7.50	15.96	80	60
Mali	5.62	11.39	17.32	29.61	63.01	69	60
Niger	0.05	0.10	0.14	0.25	0.53	65	60
Nigeria	10.60	21.48	32.64	55.80	118.59	95	59
**Sub-total: Western Africa**	**21.97**	**44.52**	**67.66**	**115.68**	**245.98**		
**Total**	**78**	**157**	**239**	**409**	**869**		

**Fig 3:**
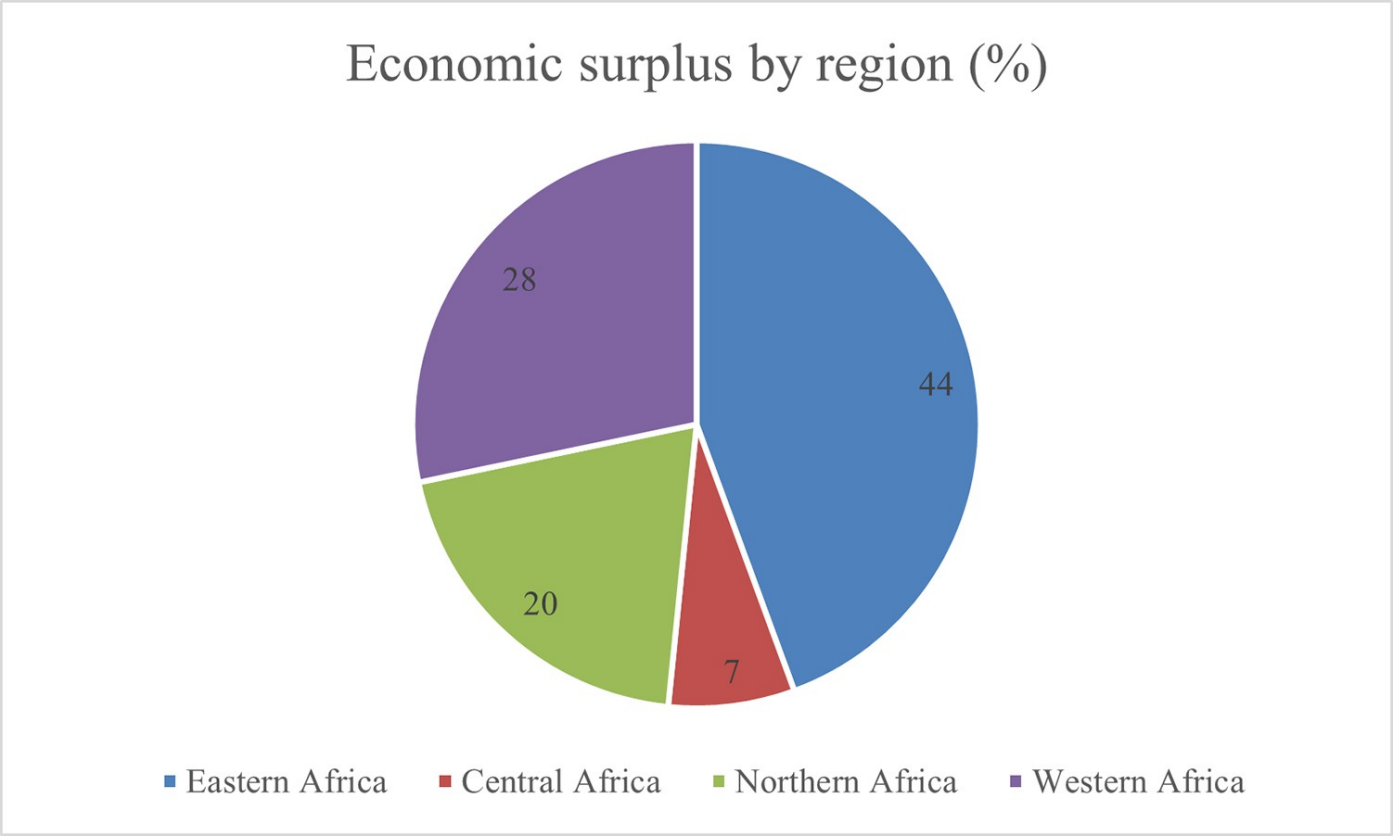
Share of economic surplus by region.

The estimated results further show that the economic surplus varies across countries. Because of the high number of meat and milk animals at risk of trypanosomiasis, 53% of the total estimated economic benefits are in Sudan, Kenya, and Nigeria. Uganda, Mali, Tanzania, Ethiopia, Zambia, and the Central African Republic takes 34% of the economic benefits. The remaining 13% of the economic surplus goes to the other countries contributing less than 3% to the total economic surplus (Table 3). Eastern African countries earned 44% (US$ 34–386 million), while 28% (US$ 22–246 million) of the total economic surplus goes to West African countries. Central Africa comes third, accruing 19% (US$ 6–63 million) of the economic surplus. The remaining 20% (US$ 16–175 million) of the economic surplus goes to Sudan, which is the only country with a significant trypanosomiasis problem in North Africa (Fig 3).

Compared to the economic losses associated with trypanosomiasis in many countries, the estimated economic benefits of WRB are huge. For instance, Eastern African countries (Ethiopia, Kenya, Somalia, South Sudan, Sudan, and Uganda) could lose US$ 2.5 billion over twenty years [10]. If 5% of the animals in Eastern Africa adopt WRB, our estimates show that the region could save 28% of the US$ 125 million per annum average economic loss due to trypanosomiasis. With the widespread adoption of the WRB (e.g., in 50% of the animals), the estimated gains in Eastern Africa alone could increase by eleven-fold, which is much bigger than the estimated losses of US$ 125 million per annum (Table 3). The estimated benefits of the technology adoption in 5% to 50% of the animals in the countries considered ranges from USD 78 to USD 869 million. This represents 6% to 65% of total economic cost due to trypanosomiasis in SSA, 1340 million per year [3].

### Benefit-cost ratio

Table 4 presented the benefit-cost ratio calculated using Eq (5). For all countries under study, the incremental benefit is greater than the incremental cost of adopting the technology. On average, a 1 US$ investment generates US$ 9 benefits if farmers adopted the WRB in 5% of their animals. The benefit-cost ratios also vary across countries. As the adoption rate increases, the benefit-cost ratio increases. On average, it increases from about US$8.77 at a 5% adoption rate to US$ 9.83 at a 50% adoption rate. However, the benefit-cost ratio shows strong heterogeneities across countries. Mali is the least to benefit from adopting WRB, while the Republic of the Congo benefits the most. Increasing WRB adoption beyond 5% could help countries reduce the impact of trypanosomiasis, thereby higher economic surplus from meat and milk production.

**Table 4:** Estimated benefit-cost ratio (US$).

	Adoption rates				
Country	5%	10%	15%	25%	50%
Ethiopia	5.49	5.56	5.64	5.79	6.16
Kenya	9.41	9.54	9.67	9.92	10.55
Mozambique	9.61	9.74	9.87	10.14	10.79
Rwanda	6.20	6.28	6.37	6.54	6.95
Uganda	9.07	9.19	9.32	9.56	10.17
Tanzania	5.17	5.24	5.31	5.45	5.80
Zambia	14.68	14.88	15.08	15.47	16.45
**Average: Eastern Africa**	**8.52**	**8.63**	**8.75**	**8.98**	**9.55**
Cameroon	3.68	3.73	3.78	3.88	4.12
CAR	6.78	6.87	6.96	7.14	7.60
Republic of the Congo	23.03	23.34	23.64	24.26	25.79
Democratic Republic of the Congo (DRC)	12.80	12.98	13.15	13.50	14.38
**Average: Central Africa**	**11.57**	**11.73**	**11.88**	**12.19**	**12.97**
**North Africa: Sudan**	**3.24**	**3.28**	**3.33**	**3.41**	**3.62**
Burkina Faso	4.96	5.03	5.10	5.23	5.56
Ghana	17.64	17.88	18.11	18.58	19.75
Guinea	9.95	10.09	10.22	10.49	11.15
Mali	2.89	2.93	2.97	3.05	3.24
Niger	4.76	4.82	4.89	5.02	5.34
Nigeria	8.46	8.57	8.68	8.91	9.47
**Average: Western Africa**	**8.11**	**8.22**	**8.33**	**8.54**	**9.08**
**Average**	**8.77**	**8.89**	**9.00**	**9.24**	**9.83**

## Conclusions and recommendations

Evidence on the cost of diseases and the benefits of disease control technology is essential for managing the disease and promoting the control measure. Trypanosomiasis affects the economies of SSA by reducing the productivity of livestock, human labor, and animal draught power. Eradicating the disease would contribute to poverty reduction and achieving sustainable development goals. This paper estimates the economic potential of adopting a repellent collar technology, an invention to drive trypanosomiasis-transmitting tsetse fly away from animals and humans.

We estimate the economic benefits using the economic surplus model, a partial equilibrium approach. We use this approach because it requires less data than other approaches (e.g., general equilibrium models). Our estimates are limited to the direct benefits of the technology on livestock production, specifically focusing on the benefit of repellent collar technology on meat and milk productivity. We have estimated the economic surplus for 18 countries out of the 39 SSA countries due to a lack of data. In short- to long-term, SSA may generate an economic surplus of US$ 78–869 million per annum through adopting the technology. The estimated benefit-cost ratio (9:1) further justifies an investment in WRB.

Although our estimate demonstrates the importance of the technology, the study has certain caveats. First, there is the need for updated and country-specific data regarding the parameters used in the analysis. Second, because we have used a partial equilibrium approach, the indirect effects of increasing meat and milk productivity could be huge, especially in SSA, where malnutrition and food insecurity are limiting factors to human labor productivity. Third, we did not estimate the contribution of the technology on increasing the productivity of draught power and human health due to a reduction in the use of chemicals to control the disease. Fourth, we did not capture the impact of technology on crop production due to an increase in the amount of land under crop cultivation following tsetse control and its environmental services benefits due to reduced pesticide use and vegetation clearing to control the disease. These caveats are associated with data constraints and the modeling approach we used. Accounting for these indirect benefits and validating expert opinions data need additional investment. The importance of developing integrated and dynamic decision-making modeling tools is particularly appealing for evaluating repellent collar technology as a one-health approach to eradicating trypanosomiasis. This is because the technology is likely to have a lasting impact not only on humans and animals but also on the natural ecosystem. Thus, we recommend future research to update and collect representative data on direct and indirect benefits of technology and use a general equilibrium approach to assess the technology’s one health (animal health, human, health, and environmental health) impact.

## Supporting information

S1 File Data processing approach: Stata codes. (PDF)

S2 File The parameters and estimated benefits of the technology: Stata file. (DTA)

S1 Data (XLSX)
