# Supplementary material for: The potential economic benefits of controlling trypanosomiasis using waterbuck repellent blend in sub-Saharan Africa
Source: PLoS One. 2021 Jul 20;16(7):e0254558. doi: 10.1371/journal.pone.0254558 (PMC8291668; doi:10.1371/journal.pone.0254558)
Supplement: S1 File — (PDF) [file pone.0254558.s001.pdf]

```
1
2
3   /*
4
5   Zewdo Abro, Menale Kassie, Beatrice Muriithi, Daniel Masiga, and
6   Gift Wiseman (2021). "The potential economic benefits of controlling
7   trypanosomiasis using waterbuck
8   repellent blend (WRB) in sub-Saharan Africa"
9
10  Prepared by Dr. Zewdu Ayalew Abro
11  icipe, Gurdshola ILRI campus
12  Addis Ababa
13  Fogera building room number 48
14  telephone: +251913601494
15  Email zabro@icipe.org; zewduayalewabro@gmail.com
16
17  The aim of this do file is three fold:
18
19  1) it prepares the required datasets for the estimation of the
    benefits of
20  introduction of the WRB
21  2) it does also do the estimation of the benefits
22  3) it facilitates the replication of results
23  4) the last but not least, it serves as a means of communication
    between
24  the author and the rest of the research team.
25
26  The do file is prepared in various blocks:
27
28  Frist block: "Miscellaneous block" brings various datasets and clean
29  for our purpose
30  Second block: "Price block" prepares data on relevant prices
31  Thrid block:  "Production block" prepares data on livestock
32  production in the study countries and merge it with the the other
    blocks
33  Fourth block: "Parameters block" prepares the parameters based on
34  equations (1) and (2) of the manuscript
35  Fifth block: "Estimation block" estimates the benefits of the
36  technology using equations (1) and (2)
37  Six and final block: "Reports block" shows the how the results
38  reported in the manuscript were calculated
39
40  */
41
42
```

```
43  ***********Miscellaneous block open***********
44  ***********Miscellaneous block open***********
45  ***********Miscellaneous block open***********
46
47  cd "C:\Users\Admin\OneDrive - International Centre of Insect
    Physiology and Ecology (ICIPE)\Economics_of_Tsetsefly\Data"
48  //cd "C:\Users\Admin\Dropbox\icipe\Economics_of_Tsetsefly\Data"
49  //value of production and contribution of the livestock economy
50  //copy
    http://fenixservices.fao.org/faostat/static/bulkdownloads/Value_of_Prod
    uction_E_Africa.zip
51  import excel "Value_of_Production_E_Africa.xlsx", ///
52  sheet("Value_of_Production_E_Africa_NO") firstrow clear
53
54  keep if Element=="Gross Production Value (current million US$)"
55  //keep if Item=="Agriculture (PIN)"|Item=="Livestock
    (PIN)"|Item=="Crops (PIN)"
56  keep if Item=="Agriculture (PIN)"|Item=="Livestock (PIN)"
57  drop if Area=="Sudan"
58
59  foreach  v of var Y1961-Y2016 {
60  gen share_liv_`v'=`v'[_n]/`v'[_n-1]
61  label var share_liv_`v' "Share of livestock to total agricultural
    value of production for `v'"
62  }
63  keep if Item=="Livestock (PIN)"
64
65  egen cont_liv_1=rowmean(share_liv_Y2013 share_liv_Y2014
    share_liv_Y2015 share_liv_Y2016)
66  label var cont_liv_1 "Contribution of livestock to total agricultural
    value of production% (2013-2016)"
67
68  egen cont_liv_2=rowmean(share_liv_Y1991-share_liv_Y2016)
69  label var cont_liv_2 "Contribution of livestock to total agricultural
    value of production% (1991-2016)"
70
71  drop Y1961-share_liv_Y2016
72   rename Area area
73   rename AreaCode areacode
74
75
76  tempfile Livestock_contribution
77  save "`Livestock_contribution'"
78
79  //Protein supply by country
```

```
import excel "FoodSupply_LivestockFish_E_All_Data.xlsx" ///
, sheet("FoodSupply_LivestockFish_E_All_") firstrow clear
keep if Element=="Protein supply quantity (g/capita/day)"
keep if Item=="Bovine Meat"|Item=="Mutton & Goat Meat"|Item=="Milk, Whole"
duplicates report AreaCode Area Item

drop Y1961F Y1962F Y1963F Y1964F Y1965F Y1966F Y1967F Y1968F Y1969F Y1970F Y1971F  ///
Y1972F Y1973F Y1974F Y1975F Y1976F Y1977F Y1978F Y1979F Y1980F Y1981F Y1982F Y1983F ///
Y1984F Y1985F Y1986F Y1987F Y1988F Y1989F Y1990F Y1991F Y1992F Y1993F Y1994F Y1995F ///
Y1996F Y1997F Y1998F Y1999F Y2000F Y2001F Y2002F Y2003F Y2004F Y2005F Y2006F Y2007F ///
Y2008F Y2009F Y2010F Y2011F Y2012F Y2013F
egen psq=rowmean(Y2009 Y2010 Y2011 Y2012 Y2013)
label var psq "Protein supply quantity (g/capita/day)"
preserve
rename psq milk_psq
label var milk_psq "Milk protein supply quantity (g/capita/day)"
keep AreaCode Area Item milk_psq
keep if Item=="Milk, Whole"
tempfile milk_protein
save "`milk_protein'"
restore
keep AreaCode Area Item psq
drop if Item=="Milk, Whole"
rename psq meat_psq
collapse (sum) meat_psq, by(AreaCode Area)
label var meat_psq "Meat protein supply quantity (g/capita/day)"

merge 1:1 AreaCode Area using "`milk_protein'", keepusing(milk_psq)
drop _merge
rename AreaCode areacode
rename Area area
tempfile meat_milk_protein
save "`meat_milk_protein'"

**********Miscellaneous block closed**********
**********Miscellaneous block closed**********
**********Miscellaneous block closed**********


**********Price block**********
```

```
120  ***********Price block***********
121  ***********Price block***********
122
123  //PPP-deflator
124  //copy
     http://api.worldbank.org/v2/en/indicator/PA.NUS.PPP?downloadformat=exce
     l
125  //before I used the data, I slightly add a country called Reunion
     using Madagascar's
126  //info and also created new variables
127  //also for western Sahara I used the values of Moroco
128  import excel "PPP conversion factor,_GDP_(LCU per
     international_$).xlsx", sheet("Data") firstrow clear
129  keep if Africa==1
130  egen ppp_def=rowmean(y2013 y2014 y2015 y2016 y2017)
131  label var ppp_def "Average PPP deflator 2013-2017 (LCU/$)"
132  drop y1960-y2018
133  tempfile PPP_deflator
134  save "`PPP_deflator'"
135
136  //exchange rate
137  //copy
     http://fenixservices.fao.org/faostat/static/bulkdownloads/Exchange_rate
     _E_Africa.zip
138  import excel "Exchange_rate_E_Africa_NOFLAG.xlsx", sheet(
     "Exchange_rate_E_Africa_NOFLAG") firstrow clear
139  egen exchange_rate=rowmean(Y2013 Y2014 Y2015 Y2016 Y2017)
140  label var exchange_rate "Average Exchange rate 2013-2017 (LCU/$)"
141  rename AreaCode areacode
142  drop Y1970-Y2018
143  tempfile exchange_rate
144  save "`exchange_rate'"
145
146
147  //Average producer prices of meat and milk
148  //copy
     http://fenixservices.fao.org/faostat/static/bulkdownloads/Prices_E_Afri
     ca.zip, replace
149  //unzipfile  Production_LivestockPrimary_E_Africa.zip, replace
150
151  import excel "Prices_E_Africa.csv.xlsx", sheet("Prices_E_Africa")
     firstrow clear
152
153  drop Y1991F Y1992F Y1993F Y1994F Y1995F Y1996F Y1997F Y1998F Y1999F
     Y2000F ///
```

```
Y2001F Y2002F Y2003F Y2004F Y2005F Y2006F Y2007F Y2008F Y2009F Y2010F ///
Y2011F Y2012F Y2013F  Y2014F Y2015F Y2016F Y2017F
keep if ElementCode==5532 //Producer Price (USD/tonne)
//keep if Element=="Producer Price (LCU/tonne)"

gen type=strpos(Item, "Meat")
gen typemilk=strpos(Item, "Milk")
replace type=1 if typemilk==1
drop if type==0
drop typemilk
drop type
gen live=strpos(Item, "live")
drop if live!=0
drop live
drop if Item=="Meat, bird nes"|Item=="Meat, chicken"|Item=="Meat, duck"|Item=="Meat, goose and guinea fowl" ///
|Item=="Meat, rabbit"|Item=="Meat, turkey"
gen meat_milk=strpos(Item, "Meat")
gen typemilk=strpos(Item, "Milk")
replace meat_milk=2 if typemilk==1
drop typemilk
drop if Area=="Ethiopia PDR"|Area=="Sudan (former)"
collapse (mean) Y1991-Y2017, by (Area AreaCode meat_milk)

//replace the missing values by the long term average growth rate of prices.
gen Y1992c=(Y1992-Y1991)/Y1991
gen Y1993c=(Y1993-Y1992)/Y1992
gen Y1994c=(Y1994-Y1993)/Y1993
gen Y1995c=(Y1995-Y1994)/Y1994
gen Y1996c=(Y1996-Y1995)/Y1995
gen Y1997c=(Y1997-Y1996)/Y1996
gen Y1998c=(Y1998-Y1997)/Y1997
gen Y1999c=(Y1999-Y1998)/Y1998
gen Y2000c=(Y2000-Y1999)/Y1999
gen Y2001c=(Y2001-Y2000)/Y2000
gen Y2002c=(Y2002-Y2001)/Y2001
gen Y2003c=(Y2003-Y2002)/Y2002
gen Y2004c=(Y2004-Y2003)/Y2003
gen Y2005c=(Y2005-Y2004)/Y2004
gen Y2006c=(Y2006-Y2005)/Y2005
gen Y2007c=(Y2007-Y2006)/Y2006
gen Y2008c=(Y2008-Y2007)/Y2007
gen Y2009c=(Y2009-Y2008)/Y2008
```

```
196   gen Y2010c=(Y2010-Y2009)/Y2009
197   gen Y2011c=(Y2011-Y2010)/Y2010
198   gen Y2012c=(Y2012-Y2011)/Y2011
199   gen Y2013c=(Y2013-Y2012)/Y2012
200   gen Y2014c=(Y2014-Y2013)/Y2013
201   gen Y2015c=(Y2015-Y2014)/Y2014
202   gen Y2016c=(Y2016-Y2015)/Y2015
203   gen Y2017c=(Y2017-Y2016)/Y2016
204   egen price_growth_rate=rowmean (Y1992c-Y2017c)
205   egen price_growth_rate1=mean(price_growth_rate)
206   replace price_growth_rate=price_growth_rate1 if price_growth_rate==.
207   drop price_growth_rate1
208   replace price_growth_rate=price_growth_rate*100
209   label var price_growth_rate "Long term average price growth rate (%)
      1991-2017"
210   drop Y1992c-Y2017c
211   replace Y1992=Y1991+(price_growth_rate/100)*Y1991 if  Y1992==.
212   replace Y1993=Y1992+(price_growth_rate/100)*Y1992 if  Y1993==.
213   replace Y1994=Y1993+(price_growth_rate/100)*Y1993 if  Y1994==.
214   replace Y1995=Y1994+(price_growth_rate/100)*Y1994 if  Y1995==.
215   replace Y1996=Y1995+(price_growth_rate/100)*Y1995 if  Y1996==.
216   replace Y1997=Y1996+(price_growth_rate/100)*Y1996 if  Y1997==.
217   replace Y1998=Y1997+(price_growth_rate/100)*Y1997 if  Y1998==.
218   replace Y1999=Y1998+(price_growth_rate/100)*Y1998 if  Y1999==.
219   replace Y2000=Y1999+(price_growth_rate/100)*Y1999 if  Y2000==.
220   replace Y2001=Y2000+(price_growth_rate/100)*Y2000 if  Y2001==.
221   replace Y2002=Y2001+(price_growth_rate/100)*Y2001 if  Y2002==.
222   replace Y2003=Y2002+(price_growth_rate/100)*Y2002 if  Y2003==.
223   replace Y2004=Y2003+(price_growth_rate/100)*Y2003 if  Y2004==.
224   replace Y2005=Y2004+(price_growth_rate/100)*Y2004 if  Y2005==.
225   replace Y2006=Y2005+(price_growth_rate/100)*Y2005 if  Y2006==.
226   replace Y2007=Y2006+(price_growth_rate/100)*Y2006 if  Y2007==.
227   replace Y2008=Y2007+(price_growth_rate/100)*Y2007 if  Y2008==.
228   replace Y2009=Y2008+(price_growth_rate/100)*Y2008 if  Y2009==.
229   replace Y2010=Y2009+(price_growth_rate/100)*Y2009 if  Y2010==.
230   replace Y2011=Y2010+(price_growth_rate/100)*Y2010 if  Y2011==.
231   replace Y2012=Y2011+(price_growth_rate/100)*Y2011 if  Y2012==.
232   replace Y2013=Y2012+(price_growth_rate/100)*Y2012 if  Y2013==.
233   replace Y2014=Y2013+(price_growth_rate/100)*Y2013 if  Y2014==.
234   replace Y2015=Y2014+(price_growth_rate/100)*Y2014 if  Y2015==.
235   replace Y2016=Y2015+(price_growth_rate/100)*Y2015 if  Y2016==.
236   replace Y2017=Y2016+(price_growth_rate/100)*Y2016 if  Y2017==.
237   drop price_growth_rate
238   egen price=rowmean(Y2013 Y2014 Y2015 Y2016 Y2017)
239   label var price "Average producer prices 2000-2017"
```

```
240   drop Y1991-Y2017
241   reshape wide price, i(AreaCode) j(meat_milk)
242   rename Area area
243   rename AreaCode areacode
244   rename price1 meat_price
245   rename price2 milk_price
246   label var meat_price "Average producer price of meat 2000-2017
      (usd/tonne)"
247   label var milk_price "Average producer price of milk 2000-2017
      (usd/tonne)"
248
249   //percentage of livestock in tsetse fly infested areas
250   gen countrygroup=.
251   replace countrygroup=5101 if areacode==29
252   replace countrygroup=5101 if areacode==45
253   replace countrygroup=5101 if areacode==72
254   replace countrygroup=5101 if areacode==178
255   replace countrygroup=5101 if areacode==238
256   replace countrygroup=5101 if areacode==62
257   replace countrygroup=5101 if areacode==114
258   replace countrygroup=5101 if areacode==129
259   replace countrygroup=5101 if areacode==130
260   replace countrygroup=5101 if areacode==137
261   replace countrygroup=5101 if areacode==270
262   replace countrygroup=5101 if areacode==144
263   replace countrygroup=5101 if areacode==182
264   replace countrygroup=5101 if areacode==184
265   replace countrygroup=5101 if areacode==196
266   replace countrygroup=5101 if areacode==201
267   replace countrygroup=5101 if areacode==277
268   replace countrygroup=5101 if areacode==226
269   replace countrygroup=5101 if areacode==215
270   replace countrygroup=5101 if areacode==251
271   replace countrygroup=5101 if areacode==181
272   replace countrygroup=5102 if areacode==7
273   replace countrygroup=5102 if areacode==32
274   replace countrygroup=5102 if areacode==37
275   replace countrygroup=5102 if areacode==39
276   replace countrygroup=5102 if areacode==46
277   replace countrygroup=5102 if areacode==250
278   replace countrygroup=5102 if areacode==61
279   replace countrygroup=5102 if areacode==74
280   replace countrygroup=5102 if areacode==193
281   replace countrygroup=5101 if areacode==276
282   replace countrygroup=5104 if areacode==20
```

```
283   replace countrygroup=5104 if areacode==209
284   replace countrygroup=5104 if areacode==122
285   replace countrygroup=5104 if areacode==147
286   replace countrygroup=5104 if areacode==202
287   replace countrygroup=5104 if areacode==209
288   replace countrygroup=5105 if areacode==53
289   replace countrygroup=5105 if areacode==233
290   replace countrygroup=5105 if areacode==35
291   replace countrygroup=5105 if areacode==107
292   replace countrygroup=5105 if areacode==75
293   replace countrygroup=5105 if areacode==81
294   replace countrygroup=5105 if areacode==90
295   replace countrygroup=5105 if areacode==175
296   replace countrygroup=5105 if areacode==123
297   replace countrygroup=5105 if areacode==133
298   replace countrygroup=5105 if areacode==136
299   replace countrygroup=5105 if areacode==158
300   replace countrygroup=5105 if areacode==159
301   replace countrygroup=5105 if areacode==187
302   replace countrygroup=5105 if areacode==195
303   replace countrygroup=5105 if areacode==197
304   replace countrygroup=5105 if areacode==217
305   replace countrygroup=5103 if areacode==4
306   replace countrygroup=5103 if areacode==59
307   replace countrygroup=5103 if areacode==124
308   replace countrygroup=5103 if areacode==143
309   replace countrygroup=5103 if areacode==276
310   replace countrygroup=5103 if areacode==206
311   replace countrygroup=5103 if areacode==206
312   replace countrygroup=5103 if areacode==222
313   replace countrygroup=5103 if areacode==205
314   merge 1:1 areacode using "`PPP_deflator'", keepusing (ppp_def)
315   drop if  _merge==2
316   drop _merge
317   merge 1:1 areacode using "`exchange_rate'", keepusing (exchange_rate)
318   drop if  _merge==2
319   drop _merge
320
321
322   tempfile prices
323   save "`prices'"
324
325   ************Price block closed************
326   ************Price block closed************
327   ************Price block closed************
```

```
328
329   ***********Production block***********
330   ***********Production block***********
331   ***********Production block***********
332
333   //Cattle population in Africa
334   //copy
      http://fenixservices.fao.org/faostat/static/bulkdownloads/Production_Li
      vestock_E_Africa.zip
335   import excel "Production_Livestock_E_Africa.xlsx", sheet(
      "Production_Livestock_E_Africa_N") firstrow clear
336   drop if Item=="Beehives"|Item=="Cattle and Buffaloes"|Item=="Chickens"
      |Item=="Ducks"|Item=="Geese and guinea fowls" ///
337   |Item=="Pigeons, other birds"|Item=="Poultry Birds"|Item=="Rabbits
      and hares"|Item=="Sheep and Goats"|Item=="Turkeys"
338
339   collapse (sum) Y1961-Y2016, by (Area AreaCode Element ElementCode Unit)
340   egen stock=rowmean(Y2013 Y2014 Y2015 Y2016)
341   label var stock "Average number of stock of  animals 2013-2017"
342   drop Y1961-Y2016
343   rename AreaCode areacode
344   rename Area area
345   tempfile stock_of_animals
346   save "`stock_of_animals'"
347
348
349   /*Live animals could be exported
350   estimates of meat from export of live animals should be accounted
351   */
352   //copy
      http://fenixservices.fao.org/faostat/static/bulkdownloads/Trade_LiveAni
      mals_E_Africa.zip, replace
353   import excel "Trade_LiveAnimals_E_Africa.csv.xlsx", sheet(
      "Trade_LiveAnimals_E_Africa") firstrow clear
354   drop Y1961F Y1962F Y1963F Y1964F Y1965F Y1966F Y1967F Y1968F Y1969F
      Y1970F Y1971F  ///
355   Y1972F Y1973F Y1974F Y1975F Y1976F Y1977F Y1978F Y1979F Y1980F Y1981F
      Y1982F Y1983F ///
356   Y1984F Y1985F Y1986F Y1987F Y1988F Y1989F Y1990F Y1991F Y1992F Y1993F
      Y1994F Y1995F ///
357   Y1996F Y1997F Y1998F Y1999F Y2000F Y2001F Y2002F Y2003F Y2004F Y2005F
      Y2006F Y2007F ///
358   Y2008F Y2009F Y2010F Y2011F Y2012F Y2013F Y2014F Y2015F Y2016F
359   keep if Item=="Asses"|Item=="Buffaloes"|Item=="Camelids, other"|Item==
      "Camels"|Item=="Cattle" ///
```

```
|Item=="Goats"|Item=="Horses"|Item=="Mules"|Item=="Pigs"
drop if Element=="Import Quantity"|Element=="Import Value"
drop if Area=="Ethiopia PDR"|Area=="Sudan (former)"
collapse (sum) Y1961-Y2016, by (Area AreaCode Element ElementCode Unit)
egen ela=rowmean(Y2013 Y2014 Y2015 Y2016)
label var ela "Average number of export of live animals 2000-2016 (tonnes)"
drop Y1961-Y2016

preserve
keep if Element=="Export Quantity"
rename ela ela_q
label var ela_q "Average number of export of live animals 2000-2016"
tempfile liveanimals_exportq
save "`liveanimals_exportq'"
restore
drop if Element=="Export Quantity"
rename ela ela_v
replace ela_v=ela_v*1000
label var ela_v "Average value of number of live animals 2000-2016 (USD)"
merge 1:1 AreaCode using "`liveanimals_exportq'", keepusing(ela_q)
tempfile liveanimals_exportq
save "`liveanimals_exportq'"
drop ElementCode Element Unit _merge
rename AreaCode areacode
rename Area area
order areacode area ela_q ela_v
tempfile live_animals_export
save "`live_animals_export'"

//raw milk could be exported
//copy http://fenixservices.fao.org/faostat/static/bulkdownloads/Trade_Crops_Livestock_E_Africa.zip, replace
import excel "Trade_Crops_Livestock_E_Africa.xlsx", sheet("Trade_Crops_Livestock_E_Africa") firstrow clear
drop Y1961F Y1962F Y1963F Y1964F Y1965F Y1966F Y1967F Y1968F Y1969F Y1970F Y1971F  ///
Y1972F Y1973F Y1974F Y1975F Y1976F Y1977F Y1978F Y1979F Y1980F Y1981F Y1982F Y1983F ///
Y1984F Y1985F Y1986F Y1987F Y1988F Y1989F Y1990F Y1991F Y1992F Y1993F Y1994F Y1995F ///
Y1996F Y1997F Y1998F Y1999F Y2000F Y2001F Y2002F Y2003F Y2004F Y2005F Y2006F Y2007F ///
```

```
Y2008F Y2009F Y2010F Y2011F Y2012F Y2013F Y2014F Y2015F Y2016F
keep if Element=="Export Quantity"|Element=="Export Value"
gen typemilk=strpos(Item, "Milk")
drop if typemilk==0
drop typemilk
keep if Item=="Milk Fresh"
collapse (sum) Y1961-Y2016, by (Area AreaCode Element ElementCode Unit)
egen emilk=rowmean(Y2013 Y2014 Y2015 Y2016)
label var emilk "Average export of milk 2000-2016 (tonnes)"
drop Y1961-Y2016
drop if Area=="Ethiopia PDR"|Area=="Sudan (former)"

preserve
keep if Element=="Export Quantity"
rename emilk emilk_q
label var emilk_q "Average quantity of export of milk 2000-2016"
tempfile milk_exportq
save "`milk_exportq'"
restore

drop if Element=="Export Quantity"
rename emilk emilk_v
replace emilk_v=emilk_v*1000
label var emilk_v "Average value of export of fresh milk 2000-2016
(usd)"
merge 1:1 AreaCode using "`milk_exportq'", keepusing(emilk_q)
drop _merge
order AreaCode Area ElementCode Element Unit emilk_q emilk_v
rename AreaCode areacode
rename Area area
tempfile milk_export
save "`milk_export'"

//domestic meat and milk produced and consumed

//copy
http://fenixservices.fao.org/faostat/static/bulkdownloads/Production_Li
vestockPrimary_E_Africa.zip Production_LivestockPrimary_E_Africa.zip,
replace
//unzipfile  Production_LivestockPrimary_E_Africa.zip, replace

//number of producing Animals/Slaughtered
import delimited Production_LivestockPrimary_E_Africa_NOFLAG.csv, clear
keep if element=="Producing Animals/Slaughtered"
keep if item=="Meat, ass"|item=="Meat, buffalo"|item=="Meat, camel"|
```

```
    item=="Meat, cattle" ///
437 |item==" Meat, goat"|item=="Meat, horse"|item=="Meat, pig"|item==
    "Meat, sheep"
438 duplicates report area item
439 collapse (sum) y1961-y2017, by (area areacode)
440 egen no_killed_animals=rowmean(y2013 y2014 y2015 y2016 y2017)
441 label var no_killed_animals "Average number of slaughtered animals
    2000-2017"
442 drop y1961-y2017
443 tempfile no_killed_animals
444 save "`no_killed_animals'"
445
446 //number of milk animals
447 import delimited Production_LivestockPrimary_E_Africa_NOFLAG.csv, clear
448 gen type=strrpos(item, "Total")
449 tab item type
450 keep if type>0
451 drop if element=="Yield"
452 drop type
453 tab item itemcode
454 tab element elementcode
455 tab item element
456 tab item unit
457 keep if element=="Milk Animals"
458 egen no_milk_animals=rowmean(y2013 y2014 y2015 y2016 y2017)
459 label var no_milk_animals "Average number of milk animals 2000-2017"
460 drop y1961-y2017
461 tempfile no_milk_animals
462 save "`no_milk_animals'"
463
464 //Livestock population
465 //copy
    http://fenixservices.fao.org/faostat/static/bulkdownloads/Production_Li
    vestock_E_Africa.zip
466 import excel "Production_Livestock_E_Africa_NOFLAG.xlsx", sheet(
    "Production_Livestock_E_Africa_N") firstrow clear
467 keep if Item=="Asses"|Item=="Buffaloes"|Item=="Camels"|Item=="Cattle"|
    Item=="Goats" ///
468 |Item=="Horses"|Item=="Mules"|Item=="Pigs"|Item=="Sheep"
469 collapse (sum) Y1961-Y2017, by (Area AreaCode)
470 drop if Area=="Ethiopia PDR"|Area=="Sudan (former)"
471 egen num_animals=rowmean(Y2013 Y2014 Y2015 Y2016 Y2017)
472 label var num_animals "Average livestock population 2000-2017"
473 drop Y1961-Y2017
474 rename AreaCode areacode
```

```
rename Area area
tempfile number_animals
save "`number_animals'"

//Productivity of meat and milk

import delimited Production_LivestockPrimary_E_Africa_NOFLAG.csv, clear
keep if element=="Yield" & item=="Milk,Total"
egen milk_yield_fao=rowmean(y2013 y2014 y2015 y2016 y2017)
replace milk_yield_fao=milk_yield_fao/10000
label var milk_yield_fao "Average milk yield 2000-2017 (tonnes/ani)"
drop y1961-y2017
tempfile milk_yield_FAO
save "`milk_yield_FAO'"

import delimited Production_LivestockPrimary_E_Africa_NOFLAG.csv, clear
keep if element=="Yield/Carcass Weight"
keep if item=="Meat, ass"|item=="Meat, buffalo"|item=="Meat, camel"|
item=="Meat, cattle" ///
|item=="Meat, game"|item=="Meat, goat"|item=="Meat, horse"| ///
item=="Meat, nes"|item=="Meat, pig"|item=="Meat, sheep"
collapse (mean) y1961-y2017, by (area areacode)
egen meat_yield_fao=rowmean(y2013 y2014 y2015 y2016 y2017)
replace meat_yield_fao=meat_yield_fao/10000
label var meat_yield_fao "Average meat yield 2000-2017 (tonnes/ani)"
drop y1961-y2017
tempfile meat_yield_FAO
save "`meat_yield_FAO'"


//Production of milk
import delimited Production_LivestockPrimary_E_Africa_NOFLAG.csv, clear
gen type=strrpos(item, "Total")
tab item type
keep if type>0
drop if element=="Yield"
drop type
tab item itemcode
tab element elementcode
tab item element
tab item unit
keep if item=="Milk,Total"
keep if element=="Production"
egen milk_prod=rowmean(y2013 y2014 y2015 y2016 y2017)
label var milk_prod "Average milk production 2000-2017 (tonnes)"
```

```
519  drop y1961-y2017
520  tempfile milk_production
521  save "`milk_production'"
522
523  //Production of meat
524  import delimited Production_LivestockPrimary_E_Africa_NOFLAG.csv, clear
525  tab item itemcode
526  tab element elementcode
527  tab item element
528  tab item unit
529  keep if element=="Production"
530  keep if item=="Meat, ass"|item=="Meat, buffalo"|item=="Meat, camel"|
     item=="Meat, cattle" ///
531  |item=="Meat, game"|item=="Meat, goat"|item=="Meat, horse"| ///
532  item=="Meat, nes"|item=="Meat, pig"|item=="Meat, sheep"
533  collapse (sum) y1961-y2017, by (area areacode)
534  egen meat_prod=rowmean(y2013 y2014 y2015 y2016 y2017)
535  label var meat_prod "Average meat production 2000-2017 (tonnes)"
536  drop y1961-y2017
537  tempfile meat_production
538  save "`meat_production'"
539  merge m:1 area using "`no_killed_animals'" , keepusing(
     no_killed_animals)
540  drop if _merge==2
541  drop _merge
542  gen meat_yield=meat_prod/no_killed_animals
543  label var meat_yield "Producitvity of meat (tonne/animal)"
544  merge m:1 area using "`milk_production'", keepusing(milk_prod)
545  drop _merge
546  merge m:1 area using "`no_milk_animals'", keepusing(no_milk_animals)
547  drop _merge
548  gen milk_yield=milk_prod/no_milk_animals
549  label var milk_yield "Producitvity of milk (tonne/animal)"
550  drop if area=="Ethiopia PDR"|area=="Sudan (former)"
551
552  merge 1:1 areacode using "`prices'", keepusing (meat_price milk_price)
553  drop _merge
554
555  merge 1:1 areacode using "`PPP_deflator'", keepusing (ppp_def)
556  drop _merge
557
558  merge 1:1 areacode using "`live_animals_export'" , keepusing (ela_q
     ela_v)
559  drop _merge
560
```

```
561   merge 1:1 areacode using "`milk_export'", keepusing (emilk_q emilk_v)
562   drop _merge
563
564   merge 1:1 areacode using "`number_animals'", keepusing (num_animals)
565   drop if _merge==2
566   drop _merge
567
568   merge 1:1 areacode using "`milk_yield_FAO'", keepusing (milk_yield_fao)
569   drop if _merge==2
570   drop _merge
571
572   merge 1:1 areacode using "`meat_yield_FAO'", keepusing (meat_yield_fao)
573   drop if _merge==2
574   drop _merge
575
576   merge 1:1 areacode using "`Livestock_contribution'" , keepusing (
      cont_liv_1 cont_liv_2)
577   drop if _merge==2
578   drop _merge
579
580   merge 1:1 areacode using "`stock_of_animals'", keepusing (stock)
581   drop if _merge==2
582   drop _merge
583
584   merge 1:1 areacode using "`meat_milk_protein'", keepusing(meat_psq
      milk_psq)
585   drop if _merge==2
586   drop _merge
587
588   //percentage of livestock in tsetse fly infested areas
589   gen countrygroup=.
590   replace countrygroup=5101 if areacode==29
591   replace countrygroup=5101 if areacode==45
592   replace countrygroup=5101 if areacode==72
593   replace countrygroup=5101 if areacode==178
594   replace countrygroup=5101 if areacode==238
595   replace countrygroup=5101 if areacode==62
596   replace countrygroup=5101 if areacode==114
597   replace countrygroup=5101 if areacode==129
598   replace countrygroup=5101 if areacode==130
599   replace countrygroup=5101 if areacode==137
600   replace countrygroup=5101 if areacode==270
601   replace countrygroup=5101 if areacode==144
602   replace countrygroup=5101 if areacode==182
603   replace countrygroup=5101 if areacode==184
```

```
604   replace countrygroup=5101 if areacode==196
605   replace countrygroup=5101 if areacode==201
606   replace countrygroup=5101 if areacode==277
607   replace countrygroup=5101 if areacode==226
608   replace countrygroup=5101 if areacode==215
609   replace countrygroup=5101 if areacode==251
610   replace countrygroup=5101 if areacode==181
611   replace countrygroup=5102 if areacode==7
612   replace countrygroup=5102 if areacode==32
613   replace countrygroup=5102 if areacode==37
614   replace countrygroup=5102 if areacode==39
615   replace countrygroup=5102 if areacode==46
616   replace countrygroup=5102 if areacode==250
617   replace countrygroup=5102 if areacode==61
618   replace countrygroup=5102 if areacode==74
619   replace countrygroup=5102 if areacode==193
620   replace countrygroup=5101 if areacode==276
621   replace countrygroup=5104 if areacode==20
622   replace countrygroup=5104 if areacode==209
623   replace countrygroup=5104 if areacode==122
624   replace countrygroup=5104 if areacode==147
625   replace countrygroup=5104 if areacode==202
626   replace countrygroup=5104 if areacode==209
627   replace countrygroup=5105 if areacode==53
628   replace countrygroup=5105 if areacode==233
629   replace countrygroup=5105 if areacode==35
630   replace countrygroup=5105 if areacode==107
631   replace countrygroup=5105 if areacode==75
632   replace countrygroup=5105 if areacode==81
633   replace countrygroup=5105 if areacode==90
634   replace countrygroup=5105 if areacode==175
635   replace countrygroup=5105 if areacode==123
636   replace countrygroup=5105 if areacode==133
637   replace countrygroup=5105 if areacode==136
638   replace countrygroup=5105 if areacode==158
639   replace countrygroup=5105 if areacode==159
640   replace countrygroup=5105 if areacode==187
641   replace countrygroup=5105 if areacode==195
642   replace countrygroup=5105 if areacode==197
643   replace countrygroup=5105 if areacode==217
644   replace countrygroup=5103 if areacode==4
645   replace countrygroup=5103 if areacode==59
646   replace countrygroup=5103 if areacode==124
647   replace countrygroup=5103 if areacode==143
648   replace countrygroup=5103 if areacode==276
```

```
replace countrygroup=5103 if areacode==206
replace countrygroup=5103 if areacode==206
replace countrygroup=5103 if areacode==222
replace countrygroup=5103 if areacode==205

label define countrygroup 5101  "Eastern Africa" ///
5102    "Central Africa" 5104   "Southern Africa" ///
5105    "Western Africa" 5103   "Northern Africa"
label values countrygroup countrygroup
label var countrygroup "Regions"

gen SSA=0
replace SSA=1 if areacode==7
replace SSA=1 if areacode==53
replace SSA=1 if areacode==20
replace SSA=1 if areacode==233
replace SSA=1 if areacode==29
replace SSA=1 if areacode==35
replace SSA=1 if areacode==32
replace SSA=1 if areacode==37
replace SSA=1 if areacode==39
replace SSA=1 if areacode==45
replace SSA=1 if areacode==46
replace SSA=1 if areacode==107
replace SSA=1 if areacode==250
replace SSA=1 if areacode==72
replace SSA=1 if areacode==61
replace SSA=1 if areacode==178
replace SSA=1 if areacode==209
replace SSA=1 if areacode==238
replace SSA=1 if areacode==74
replace SSA=1 if areacode==75
replace SSA=1 if areacode==81
replace SSA=1 if areacode==90
replace SSA=1 if areacode==175
replace SSA=1 if areacode==114
replace SSA=1 if areacode==122
replace SSA=1 if areacode==123
replace SSA=1 if areacode==129
replace SSA=1 if areacode==130
replace SSA=1 if areacode==133
replace SSA=1 if areacode==136
replace SSA=1 if areacode==137
replace SSA=1 if areacode==144
replace SSA=1 if areacode==147
```

```
694   replace SSA=1 if areacode==158
695   replace SSA=1 if areacode==159
696   replace SSA=1 if areacode==184
697   replace SSA=1 if areacode==193
698   replace SSA=1 if areacode==195
699   replace SSA=1 if areacode==196
700   replace SSA=1 if areacode==197
701   replace SSA=1 if areacode==201
702   replace SSA=1 if areacode==202
703   replace SSA=1 if areacode==277
704   replace SSA=1 if areacode==276
705   replace SSA=1 if areacode==217
706   replace SSA=1 if areacode==226
707   replace SSA=1 if areacode==215
708   replace SSA=1 if areacode==251
709   replace SSA=1 if areacode==181
710   replace SSA=1 if areacode==182
711   label var SSA "1 if sub-Saharan Africa, 0 otherwise"
712
713   gen pct_animals_tsetse=8 if countrygroup==5104
714   replace pct_animals_tsetse=28 if countrygroup==5101
715   replace pct_animals_tsetse=48 if countrygroup==5105
716   replace pct_animals_tsetse=32 if countrygroup==5102
717   replace pct_animals_tsetse=0 if countrygroup==5103
718   label var pct_animals_tsetse "Percentage of animals in tstse infested
      area (%)"
719   gen try_risk_ani=num_animals*(pct_animals_tsetse/100)
720   label var try_risk_ani "Average number of animals at risk of
      trypanosomiasis"
721
722   gen pct_preva_animal_HAT_EOS=.
723   replace pct_preva_animal_HAT_EOS=8.12 if areacod==238
724   replace pct_preva_animal_HAT_EOS=6.54125 if areacod==233
725   replace pct_preva_animal_HAT_EOS=15 if areacod==32
726   replace pct_preva_animal_HAT_EOS=46.75 if areacod==37
727   replace pct_preva_animal_HAT_EOS=33.33 if areacod==46
728   replace pct_preva_animal_HAT_EOS=5 if areacod==250
729   replace pct_preva_animal_HAT_EOS=8.5 if areacod==81
730   replace pct_preva_animal_HAT_EOS=8.75 if areacod==90
731   replace pct_preva_animal_HAT_EOS=12 if areacod==114
732   replace pct_preva_animal_HAT_EOS=20 if areacod==133
733   replace pct_preva_animal_HAT_EOS=9.63636363636364 if areacod==144
734   replace pct_preva_animal_HAT_EOS=1.16666666666667 if areacod==158
735   replace pct_preva_animal_HAT_EOS=9.48333333333333 if areacod==159
736   replace pct_preva_animal_HAT_EOS=6.2 if areacod==184
```

```
replace pct_preva_animal_HAT_EOS=9 if areacod==276
replace pct_preva_animal_HAT_EOS=9.55555555555556 if areacod==215
replace pct_preva_animal_HAT_EOS=20 if areacod==226
replace pct_preva_animal_HAT_EOS=14.5833333333333 if areacod==251
label var pct_preva_animal_HAT_EOS "Experts info on prevalence rate
of HAT (%)"

gen pct_risk_animal_HAT_EOS=.
replace pct_risk_animal_HAT_EOS=20 if areacode==238 //no of cattle in
2016 was 59486667. Leta et al (2016) said 10-14 million cattle at
risk;  dis ((10000000+14000000)/2)/59486667=20%
replace pct_risk_animal_HAT_EOS=34.7186956521739 if areacode==233
replace pct_risk_animal_HAT_EOS=38.8181818181818 if areacode==32
replace pct_risk_animal_HAT_EOS=35.7272727272727 if areacode==37
replace pct_risk_animal_HAT_EOS=33.33 if areacode==46
replace pct_risk_animal_HAT_EOS=18.4736842105263 if areacode==250
replace pct_risk_animal_HAT_EOS=47.2380952380952 if areacode==81
replace pct_risk_animal_HAT_EOS=40.5 if areacode==90
replace pct_risk_animal_HAT_EOS=46.25 if areacode==114
replace pct_risk_animal_HAT_EOS=17.4761904761905 if areacode==133
replace pct_risk_animal_HAT_EOS=32.25 if areacode==144
replace pct_risk_animal_HAT_EOS=3.5 if areacode==158
replace pct_risk_animal_HAT_EOS=29.805652173913 if areacode==159
replace pct_risk_animal_HAT_EOS=37.3636363636364 if areacode==184
replace pct_risk_animal_HAT_EOS=48.9090909090909 if areacode==276
replace pct_risk_animal_HAT_EOS=35 if areacode==215
replace pct_risk_animal_HAT_EOS=28.2647058823529 if areacode==226
replace pct_risk_animal_HAT_EOS=45.5882352941176 if areacode==251
label var pct_risk_animal_HAT_EOS "Experts info on animals at risk of
HAT (%)"


************Production block closed************
************Production block closed************
************Production block closed************

************Parameters block************
************Parameters block************
************Parameters block************

//estimation of baseline meat and milk production and prices:
//we are not interested on countries of Northern Africa
//drop if countrygroup==5103

//Quantity of meat and milk production
```

```
778   egen total_meat_ani=rowtotal(no_killed_animals ela_q) //include
      exports
779   label var total_meat_ani "Number of total meat animals including
      exports"
780
781   gen Q0meat=(total_meat_ani)*(pct_risk_animal_HAT_EOS/100)*(
      pct_preva_animal_HAT_EOS/100)*(meat_yield/2) //yield is 50% lower in
      tsetse infested areas Swallow et al. (2000).
782   label var Q0meat "Average meat production by animals affected by HAT
      (tonnes)"
783   gen Q0milk=(no_milk_animals)*(pct_risk_animal_HAT_EOS/100)*(
      pct_preva_animal_HAT_EOS/100)*(milk_yield/2) //yield is 50% lower in
      tsetse infested areas Swallow et al. (2000).
784   label var Q0milk "Average milk production by animals affected by HAT
      (tonnes)"
785
786   gen price_milk_EOS=.
787   replace price_milk_EOS=360 if areacode==233
788   replace price_milk_EOS=700 if areacode==32
789   replace price_milk_EOS=2.75 if areacode==37
790   replace price_milk_EOS=1000 if areacode==46
791   replace price_milk_EOS=1500 if areacode==250
792   replace price_milk_EOS=8 if areacode==81
793   replace price_milk_EOS=10000 if areacode==90
794   replace price_milk_EOS=90 if areacode==114
795   replace price_milk_EOS=600 if areacode==133
796   replace price_milk_EOS=34.86 if areacode==144
797   replace price_milk_EOS=450 if areacode==158
798   replace price_milk_EOS=533.333333333333 if areacode==159
799   replace price_milk_EOS=200 if areacode==184
800   replace price_milk_EOS=64.5 if areacode==276
801   replace price_milk_EOS=2000 if areacode==215
802   replace price_milk_EOS=3000 if areacode==226
803   replace price_milk_EOS=5.5 if areacode==251
804   replace price_milk_EOS=price_milk_EOS/ppp_def*1000
805   label var price_milk_EOS "Price of milk in PPP"
806
807   gen price_meat_EOS=.
808   replace price_meat_EOS=1176.66666666667 if areacode==233
809   replace price_meat_EOS=2300 if areacode==32
810   replace price_meat_EOS=5.66666666666667 if areacode==37
811   replace price_meat_EOS=4166.66666666667 if areacode==46
812   replace price_meat_EOS=7000 if areacode==250
813   replace price_meat_EOS=33.3333333333333 if areacode==81
814   replace price_meat_EOS=18750 if areacode==90
```

```
replace price_meat_EOS=457.5 if areacode==114
replace price_meat_EOS=2250 if areacode==133
replace price_meat_EOS=790.193333333333 if areacode==144
replace price_meat_EOS=4000 if areacode==158
replace price_meat_EOS=1450 if areacode==159
replace price_meat_EOS=2100 if areacode==184
replace price_meat_EOS=386.666666666667 if areacode==276
replace price_meat_EOS=5500 if areacode==215
replace price_meat_EOS=13666.6666666667 if areacode==226
replace price_meat_EOS=46.6666666666667 if areacode==251
replace price_meat_EOS=price_meat_EOS/ppp_def*1000
label var price_meat_EOS "Price of meat in PPP"

egen meat_deno=total(meat_prod), by(countrygroup)
egen milk_deno=total(milk_prod), by(countrygroup)
gen meat_num=meat_prod*meat_price
egen meat_num_total=total(meat_num), by(countrygroup)
gen milk_num=milk_prod*milk_price
egen milk_num_total=total(milk_num), by(countrygroup)
gen price_meat_reg=meat_num_total/meat_deno
gen price_milk_reg=milk_num_total/milk_deno
drop meat_deno milk_deno meat_num meat_num_total milk_num
milk_num_total
label var price_meat_reg "Regional weighted average price of meat
(USD/tonne)"
label var price_milk_reg "Regional weighted average price of milk
(USD/tonne)"

//Elasticity of demand by country
//source: Muhammed et al (2013) Table 6
gen ela_dd_meat=.
replace ela_dd_meat=-0.592 if areacode==7
replace ela_dd_meat=-0.579 if areacode==53
replace ela_dd_meat=-0.587 if areacode==233
replace ela_dd_meat=-0.61 if areacode==29
replace ela_dd_meat=-0.57 if areacode==32
replace ela_dd_meat=-0.55 if areacode==35
replace ela_dd_meat=-0.591 if areacode==37
replace ela_dd_meat=-0.591 if areacode==39
replace ela_dd_meat=-0.621 if areacode==250
replace ela_dd_meat=-0.584 if areacode==46
replace ela_dd_meat=-0.578 if areacode==107
replace ela_dd_meat=-0.575 if areacode==72
replace ela_dd_meat=-0.53 if areacode==59
replace ela_dd_meat=-0.537 if areacode==61
```

```
replace ela_dd_meat=-0.601 if areacode==238
replace ela_dd_meat=-0.543 if areacode==74
replace ela_dd_meat=-0.591 if areacode==75
replace ela_dd_meat=-0.581 if areacode==81
replace ela_dd_meat=-0.588 if areacode==90
replace ela_dd_meat=-0.601 if areacode==175
replace ela_dd_meat=-0.575 if areacode==114
replace ela_dd_meat=-0.559 if areacode==122
replace ela_dd_meat=-0.608 if areacode==123
replace ela_dd_meat=-0.585 if areacode==129
replace ela_dd_meat=-0.595 if areacode==130
replace ela_dd_meat=-0.591 if areacode==133
replace ela_dd_meat=-0.579 if areacode==136
replace ela_dd_meat=-0.494 if areacode==137
replace ela_dd_meat=-0.554 if areacode==143
replace ela_dd_meat=-0.598 if areacode==144
replace ela_dd_meat=-0.546 if areacode==147
replace ela_dd_meat=-0.601 if areacode==158
replace ela_dd_meat=-0.574 if areacode==159
replace ela_dd_meat=-0.594 if areacode==184
replace ela_dd_meat=-0.569 if areacode==193
replace ela_dd_meat=-0.574 if areacode==195
replace ela_dd_meat=-0.589 if areacode==197
replace ela_dd_meat=-0.507 if areacode==202
replace ela_dd_meat=-0.561 if areacode==276
replace ela_dd_meat=-0.532 if areacode==209
replace ela_dd_meat=-0.59 if areacode==215
replace ela_dd_meat=-0.584 if areacode==217
replace ela_dd_meat=-0.522 if areacode==222
replace ela_dd_meat=-0.585 if areacode==226
replace ela_dd_meat=-0.585 if areacode==251
replace ela_dd_meat=-0.604 if areacode==181
//replace the rest by the low income average
replace ela_dd_meat=-0.564 if ela_dd_meat==.
label var ela_dd_meat "Elasticity of demand for meat"

gen ela_dd_milk=.
replace ela_dd_milk=-0.612 if areacode==7
replace ela_dd_milk=-0.599 if areacode==53
replace ela_dd_milk=-0.608 if areacode==233
replace ela_dd_milk=-0.632 if areacode==29
replace ela_dd_milk=-0.59 if areacode==32
replace ela_dd_milk=-0.568 if areacode==35
replace ela_dd_milk=-0.611 if areacode==37
replace ela_dd_milk=-0.612 if areacode==39
```

```
replace ela_dd_milk=-0.643 if areacode==250
replace ela_dd_milk=-0.605 if areacode==46
replace ela_dd_milk=-0.597 if areacode==107
replace ela_dd_milk=-0.594 if areacode==72
replace ela_dd_milk=-0.548 if areacode==59
replace ela_dd_milk=-0.556 if areacode==61
replace ela_dd_milk=-0.622 if areacode==238
replace ela_dd_milk=-0.561 if areacode==74
replace ela_dd_milk=-0.612 if areacode==75
replace ela_dd_milk=-0.601 if areacode==81
replace ela_dd_milk=-0.609 if areacode==90
replace ela_dd_milk=-0.622 if areacode==175
replace ela_dd_milk=-0.595 if areacode==114
replace ela_dd_milk=-0.578 if areacode==122
replace ela_dd_milk=-0.629 if areacode==123
replace ela_dd_milk=-0.606 if areacode==129
replace ela_dd_milk=-0.617 if areacode==130
replace ela_dd_milk=-0.611 if areacode==133
replace ela_dd_milk=-0.599 if areacode==136
replace ela_dd_milk=-0.51 if areacode==137
replace ela_dd_milk=-0.573 if areacode==143
replace ela_dd_milk=-0.619 if areacode==144
replace ela_dd_milk=-0.565 if areacode==147
replace ela_dd_milk=-0.622 if areacode==158
replace ela_dd_milk=-0.594 if areacode==159
replace ela_dd_milk=-0.615 if areacode==184
replace ela_dd_milk=-0.589 if areacode==193
replace ela_dd_milk=-0.594 if areacode==195
replace ela_dd_milk=-0.609 if areacode==197
replace ela_dd_milk=-0.525 if areacode==202
replace ela_dd_milk=-0.58 if areacode==276
replace ela_dd_milk=-0.55 if areacode==209
replace ela_dd_milk=-0.61 if areacode==215
replace ela_dd_milk=-0.604 if areacode==217
replace ela_dd_milk=-0.54 if areacode==222
replace ela_dd_milk=-0.606 if areacode==226
replace ela_dd_milk=-0.606 if areacode==251
replace ela_dd_milk=-0.625 if areacode==181
//replace the rest by the low income countries average
replace ela_dd_milk=-0.584 if ela_dd_milk==.
label var ela_dd_milk "Elasticity of demand for milk"

//Elasticity of supply by country
//Source: Alston et al. (1995): when supply elasticity estimate
//is not available Alston and colleagues suggest to use 1.
```

```
gen ela_ss_meat=0.4
label var ela_ss_meat "Meat supply elasticity"
gen ela_ss_milk=0.4
label var ela_ss_milk "Milk supply elasticity"

***********Parameters block closed***********
***********Parameters block closed***********
***********Parameters block closed***********


***********Estimation block***********
***********Estimation block***********
***********Estimation block***********


 //Figure 1: livestock contribution
tab area, sum(cont_liv_2)
tab countrygroup, sum(cont_liv_2)

drop if Q0milk==. //drop if information is missing for a given country

//Estimation of K-shift parameter

gen att_meat_y=0.50 //Kristjanson et al (1999)
label var att_meat_y "Proportionate change in meat producitvity due
to RCT"
gen att_meat_c=-1.53 //Saini et al (2017)
label var att_meat_c "Proportionate change in cost of
production(meat) due to RCT"
gen att_milk_y=0.51 //Kristjanson et al (1999)
label var att_milk_y "Proportionate change in milk producitvity due
to RCT"
gen att_milk_c=-1.53 //Saini et al (2017)
label var att_milk_c "Proportionate change in cost of production
(milk) due to RCT"

foreach i of numlist 5 10 15 25 50 {
gen k_meat`i'=((att_meat_y/ela_ss_meat)-(att_meat_c/(1+att_meat_y)))*(
`i'/100)
label var k_meat`i' "technology induced meat K-shift parameter `i'%"

gen k_milk`i'=((att_milk_y/ela_ss_milk)-(att_milk_c/(1+att_milk_y)))*(
`i'/100)
label var k_milk`i' "technology induced milk K-shift parameter `i'%"

```

```
//relative price change: Z

gen z_meat`i'=(k_meat`i'*ela_ss_meat)/(ela_ss_meat+abs(ela_dd_meat))
label var z_meat`i' "Relative price changes of meat `i'%"
gen z_milk`i'=(k_milk`i'*ela_ss_milk)/(ela_ss_milk+abs(ela_dd_milk))
label var z_milk`i' "Relative price changes of milk `i'%"

//Producer surplus

gen changePS_meat`i'=(price_meat_reg*Q0meat)*(k_meat`i'-z_meat`i')*(1+
0.5*z_meat`i'*abs(ela_dd_meat))
label var changePS_meat`i' "Meat producer surplus (USD) `i'%"
gen changePS_milk`i'=(price_milk_reg*Q0milk)*(k_milk`i'-z_milk`i')*(1+
0.5*z_milk`i'*abs(ela_dd_milk))
label var changePS_milk`i' "Milk producer surplus (USD) `i'%"

//Consumer surplus

gen changeCS_meat`i'=(price_meat_reg*Q0meat*z_meat`i')*(1+0.5*z_meat
`i'*abs(ela_dd_meat))
label var changeCS_meat`i' "Meat consumer surplus (USD) `i'%"
gen changeCS_milk`i'=(price_milk_reg*Q0milk*z_milk`i')*(1+0.5*z_milk
`i'*abs(ela_dd_milk))
label var changeCS_milk`i' "Milk consumer surplus (USD) `i'%"

//totals
egen totalPS_mt_mlk`i'=rowtotal(changePS_meat`i' changePS_milk`i')
label var totalPS_mt_mlk`i' "Total meat and milk producer surplus
(USD) `i'%"
egen totalCS_mt_mlk`i'=rowtotal(changeCS_meat`i' changeCS_milk`i')
label var totalCS_mt_mlk`i' "Total meat and milk consumer surplus
(USD) `i'%"

egen totalES_meat`i'=rowtotal(changePS_meat`i' changeCS_meat`i')
label var totalES_meat`i' "Total meat economic surplus (USD) `i'%"
egen totalES_milk`i'=rowtotal(changePS_milk`i' changeCS_milk`i')
label var totalES_milk`i' "Total milk economic surplus (USD) `i'%"
egen totalES`i'=rowtotal(totalES_meat`i' totalES_milk`i')
label var totalES`i' "Total meat and milk economic surplus (USD) `i'%"
gen share_PS`i'=totalPS_mt_mlk`i'/totalES`i'*100
label var share_PS`i' "Contribution of producer surplus to total
economic surplus (USD) `i'%"

egen totalESSA`i'=total(totalES`i')
label var totalESSA`i' "SSA total economic surplus (USD) `i'%"
```

```
gen share_EScountry`i'=totalES`i'/totalESSA`i'*100
label var share_EScountry`i' "Contribution of country to total SSA economic surplus (USD) `i'%"

}

//Benefit cost ratio of the repellent collar technology
//total_meat_ani no_milk_animals
//if the technology is applied to every animal in the herd, the cost of the technology will be estimated as follows
//first generate the proportion of animals at risk of tsetse fly and trypanosomiasis
gen stock_at_risk=(total_meat_ani+no_milk_animals)*(pct_risk_animal_HAT_EOS/100)*(pct_preva_animal_HAT_EOS/100)
label var stock_at_risk "Number of meat and milk animals at risk of trypanosomiasis"

//cost per unit of the RCT per month is 3.84 USD (Saini et al (2017))
gen unit_cost_rct=2.5*12+2.5*12*0.05 //cost of the RCT per year
label var unit_cost_rct "Unit cost of acquiring the RCT (animal/year)"

//adoption rate is at 5, 10 and 15%
foreach i of numlist 5 10 15 25 50 {
gen cost_of_RCT`i'=stock_at_risk*(`i'/100)*unit_cost_rct
label var cost_of_RCT`i' "Cost per year (USD) at `i' adoption of RCT"
}

//calculate the benefit cost ratio

gen BCR5=totalES5/cost_of_RCT5
gen BCR10=totalES10/cost_of_RCT10
gen BCR15=totalES15/cost_of_RCT15
gen BCR25=totalES25/cost_of_RCT25
gen BCR50=totalES50/cost_of_RCT50

foreach i of numlist 5 10 15 25 50 {
label var BCR`i' "Benefit cost ratio at `i'% adoption of RCT"
}

save "The economics_tsetsefly_control_final", replace

**********Estimates block closed**********
**********Estimates block closed**********
**********Estimates block closed**********
```

```
1064
1065    ***********Reports block open***********
1066    ***********Reports block open***********
1067    ***********Reports block open***********
1068
1069
1070    //Table 1: parameters of the economic surplus
1071
1072
1073    estpost sum total_meat_ani no_milk_animals pct_preva_animal_HAT_EOS
        pct_risk_animal_HAT_EOS ///
1074    meat_yield milk_yield Q0meat Q0milk ///
1075    ela_dd_meat ela_dd_milk ela_ss_meat ela_ss_milk ///
1076    price_meat_reg price_milk_reg
1077    esttab, cell("mean(fmt(%12.0g)) sd(fmt(%12.0g)) min(fmt(%12.0g))
        max(fmt(%12.0g)) sum(fmt(%12.0g))") nonumber nomtitle
1078
1079    //total number of animals prevalence rate, production
1080    table area, contents(sum total_meat_ani sum no_milk_animals) format (%
        12.0g)
1081    table area, contents(mean pct_preva_animal_HAT_EOS mean
        pct_risk_animal_HAT_EOS)
1082    table area, contents(mean stock_at_risk sum Q0meat sum Q0milk) format
        (%12.0g)
1083    table area, contents(mean meat_yield sum milk_yield) format (%12.0g)
1084
1085    //Figure 2
1086
1087    foreach v of var k_meat5  k_meat10 k_meat15 k_meat25 k_meat50 ///
1088    k_milk5 k_milk10 k_milk15 k_milk25 k_milk50 {
1089    replace `v'=`v'*100
1090    }
1091    sum k_meat5  k_meat10 k_meat15 k_meat25 k_meat50 ///
1092    k_milk5 k_milk10 k_milk15 k_milk25 k_milk50
1093
1094    //Table 2 is the summary of the expert opinions survey: see the excel
        sheet
1095
1096    //Table 3
1097    gen share_meat_5=totalES_meat5/totalES5
1098    gen share_meat_10=totalES_meat10/totalES10
1099    gen share_meat_15=totalES_meat15/totalES15
1100    gen share_meat_25=totalES_meat25/totalES25
1101    gen share_meat_50=totalES_meat50/totalES50
1102
```

```
1103    table area, contents(mean share_meat_5 mean share_meat_10 mean ///
1104    share_meat_15 mean share_meat_25 mean share_meat_50) format (%12.0g)
1105
1106    table area, contents(sum totalES5 sum totalES10 sum totalES15 ///
1107    sum totalES25 sum totalES50) format (%12.0g)
1108
1109    egen total5=total(totalES5), by(countrygroup)
1110    egen total10=total(totalES10), by(countrygroup)
1111    egen total15=total(totalES15), by(countrygroup)
1112    egen total25=total(totalES25), by(countrygroup)
1113    egen total50=total(totalES50), by(countrygroup)
1114
1115    gen share_total5=total5/totalESSA5*100
1116    gen share_total10=total10/totalESSA10*100
1117    gen share_total15=total15/totalESSA15*100
1118    gen share_total25=total25/totalESSA25*100
1119    gen share_total50=total50/totalESSA50*100
1120
1121
1122    table countrygroup, c(mean share_total5 mean share_total10 mean
        share_total15 mean share_total25 mean share_total50)
1123    drop total* share_total1-share_total50
1124
1125    //Table 4
1126    table area, c(mean BCR5 mean BCR10 mean BCR15 mean BCR25 mean BCR50)
1127
1128
1129    ************Reports block closed************
1130    ************Reports block closed************
1131    ************Reports block closed************
1132
1133
```
